# Does Being In-Person Matter? Demonstrating the Feasibility and Reliability of Fully Remote Observational Data Collection

**DOI:** 10.1007/s11121-024-01706-6

**Published:** 2024-07-12

**Authors:** Sydni A. J. Basha, Qiyue Cai, Susanne Lee, Tiffany Tran, Amy Majerle, Shauna Tiede, Abigail H. Gewirtz

**Affiliations:** 1 The REACH Institute, Department of Psychology, Arizona State University, 900 S. McAllister Avenue, Suite 205, Tempe, AZ 85281, USA; 2 Paul Baerwald School of Social Work and Social Welfare, Hebrew University of Jerusalem, Jerusalem, Israel

**Keywords:** Observational research, Observational methodology, Parenting, COVID, Military families

## Abstract

Many conventional research methods employed in randomized controlled trials were not possible during the height of the COVID-19 pandemic. In particular, behavioral observations are nearly universally gathered in-person. Observational methods are valued for the rich, informative data they produce in comparison to non-observational methods and are a cornerstone of parenting and family research. COVID provided the opportunity to, and indeed necessitated, the transition to fully remote observation. However, little to no studies have investigated whether remotely collected observational data are methodologically sound. This paper assesses the feasibility of remote data collection by describing the transition between in-person and fully remote observational data collection during a Sequential, Multiple Assignment, Randomized Trial (SMART) of a parenting program that took place both before and during the pandemic. Using mixed-methods data from coders, the overall quality of video-recorded data collected both before and during COVID was examined. Coder reliability over time was assessed with intraclass correlation coefficients. Results suggest that the frequency of audio problems, the severity of visual problems, and the level of administration challenges *decreased* after transitioning to remote data collection. Additionally, coders showed good to excellent reliability coding remotely collected data, and reliability even improved on some measured tasks. Although challenges to remote data collection exist, this study demonstrated that observational data can be collected feasibly and reliably. As observational data collection is a key method to assess parenting practices, these findings should improve researcher confidence in utilizing remote observational methods in prevention science.

## Introduction

The COVID-19 pandemic disrupted the standard practices of many family-based randomized controlled trials. In particular, researchers seeking to collect observational data were tasked with transitioning from in-person to fully remote collection methods with little precedent for how to do so effectively, efficiently, and without compromising data quality. This paper describes and examines the feasibility and reliability of fully remote, technology-assisted parent–child observational data collected before the initiation of the COVID lockdown (pre-COVID; 2017–2020) and during the lockdown period once research activities were allowed to resume (peri-COVID; 2020–2022).

Observational data collection is critical to better understanding parent–child relationships and parenting. Direct observations provide a lens on behaviors of interest (encouragement, problem-solving, positive involvement, etc.) which can be identified accurately and reliably by researchers. In the context of parent–child interactions, observations allow for a direct view of the processes within the interaction as they take place. Such detail would be difficult to capture through self-report measures, as many of the behaviors of interest may be automatic, subconscious, non-verbal, and fast-moving (Capaldi & Eddy, 2005; Eddy et al., 1998; Prescott et al., 2000). Self-report measures of parenting also are likely to be affected by systematic personal biases including parental expectations, mood, and pre-existing attributions about a child (Eddy et al., 1998). For children, reporting on their parenting requires the capacity to reflect on the parent–child relationship, an advanced cognitive function that may be beyond many young children, calling into question the reliability and accuracy of such reports (Bevans et al., 2020).

Beginning as early as the 1980s, researchers at the Oregon Social Learning Center and other research institutions used structured family interaction tasks (FITs) to conduct direct observations of parent–child interactions (e.g., Foster et al., 1983; Reid & Patterson, 1989). Typically, FITs provide insight into parenting skills including family problem-solving, monitoring, discipline, positive involvement, and skill encouragement (Forgatch & DeGarmo, 1999). Interactions are recorded and then coded using a standardized and reliable Coder Impressions (CI) global coding system (e.g., Forgatch et al., 1992). FITs have been used as a gold standard for assessing change in parenting in a variety of parenting programs, particularly those of the Oregon model of family therapy, including the Family Check-Up (Dishion et al., 2003) and GenerationPMTO (formerly known as the Parent Management Training-Oregon model) family of interventions (see, e.g., Dishion et al., 2016; Gewirtz et al., 2018).

While gathering observational data on parent–child interactions is a highly robust method of assessing parenting (Hawes & Dadds, 2006), it is costly both in terms of time and money. The expense of observational data collection techniques frequently limits the number of observations that can be conducted, potentially leading to issues of low data stability (Stoolmiller et al., 2000). Traditional methods require visits to family homes or laboratory-based assessments, both of which require significant time, travel, equipment, and ecological footprint (Oliver & Pike, 2019). These methods can also be time-consuming in terms of training staff, conducting the observations, and checking inter-observer reliability (Gardner, 2000; Margolin et al., 1998). For families, assessments are time-consuming, particularly for working caregivers focused on securing basic needs and balancing work with childcare responsibilities (Narayan et al., 2012).

Given the costs associated with observational data collection, much of the examination of parenting now relies on parental self-reports. Though self-reports and observations purport to measure similar constructs, they tend to display only small to moderate associations with one another (Hendriks et al., 2018). Observational methods and self-reports have shortcomings when considered individually; however, each offers unique and critical information for understanding various psychological outcomes (Moens et al., 2018). Accordingly, applying multiple methods is ideal for achieving the clearest understanding of family processes (Dunn & Kendrick, 1980; Giusto et al., 2019). Unfortunately, resource limitations often rule out traditional home- or lab-based observational methods as a feasible approach.

The development of scalable, efficient, and effective observational assessment strategies, then, is a crucial need for parenting research, and online assessments have the potential to address the drawbacks of in-person assessments. Technology has increasingly been applied over the past decade or so to advance or complement existing approaches. Narayan et al. (2012) used a version of the *Five-Minute Speech Sample* (a measure assessing the parent–child relationship that requires the parent to talk for 5 min about the child) along with traditional observational data collection to assess parents’ critical and positive statements, negative affect, and expressions of warmth. Results suggested the brief observational tool is a potentially useful proxy for observations and far more efficient to gather and code. Oliver and Pike (2019) introduced an online observation tool called *Etch-a-Sketch Online* (which requires a parent and child to draw an image with an online etch-a-sketch) to provide a resource-efficient observation of the family home. The parent and child are assigned to one of two control dials, corresponding to vertical and horizontal movement, and instructed to cooperate to draw the image. Results of that study indicated evidence of inter-rater reliability and predictive validity; observed parenting was associated with children’s problem behavior above and beyond parental self-reports. Additionally, online methods permit the use of recording features that do not require the physical presence of research staff, eliminating the need for families to travel to the laboratory for assessment.

This article aims to build on the observational data collection literature by reporting on the development and reliability of a fully remote, online observational assessment procedure created by necessity during the COVID-19 pandemic, amid ongoing (previously in-person home-based) data collection for a randomized controlled trial. The article aims to answer two questions: (i) What is the feasibility of remotely gathering and recording observational parent–child data (e.g., how does it compare to in-person data collection with regard to barriers and facilitators?) (ii) Are coded observations gathered online as reliable as coded observations from in-person data collection?

## Method

### Participants

The sample included 290 military families from three military installations (Fort Bragg, *n* = 135; Fort Campbell, *n* = 72; and Forts Belvoir/Myer (FBM), *n* = 83). Participants were recruited on a rolling basis^1^ and consented to participate in a Sequential, Multiple Assignment, Randomized Trial (SMART) of the Adaptive Parenting Tools (ADAPT) program. Eligibility to participate was based on the following inclusion criteria: (a) at least one parent was an active duty service member at Fort Bragg, NC (now Fort Liberty), Fort Campbell, KY, Fort Belvoir, VA or Joint Base Myer-Henderson Hall, VA; (b) at least one parent had been deployed to the recent conflicts (once or more in the past 5 years for active duty, not Special Operations; two times or more in the past 3 years for Special Operations (SO)), and (c) and at least one child between the ages of 5 and 12 was living in the home. For families with more than one eligible child, a study target child was randomly selected.

Of the 290 families, 226 families were active duty, not SO, and 64 families were SO. On average, parents were 35.5 years old (*n*_father_ = 203, *M*_father_ = 35.8, *SD*_father_ = 5.4; *n*_mother_ = 280, *M*_mother_ = 35.3, *SD*_mother_ = 5.3). Most parents (61%) had an associate degree or higher, and the median family household income was $51,000–$100,000. Parents were mostly White (79.7%), followed by African American (8.8%), Asian (2.8%), Native American (1.3%), Pacific Islander (1.1%), and Other (e.g., multiracial or did not wish to specify; 6.4%). The mean age of the target child was 8.2 years (*SD* = 2.1), and about half were girls (*n* = 150, 51.7%). Significant differences were found between pre- and peri-COVID on some measures of demographic information including parent and child age, parent education, and household income, likely as a result of the later inclusion of FBM. A detailed breakdown of demographic information pre- and peri-COVID is presented in Table 1.

### Procedures

The original study protocol included in-home, in-person assessments at three time points (baseline/pre-randomization, 1-year post-baseline, and 2-year post-baseline). Assessors would travel to families’ homes for the 2.5 to 3-h visit and conduct consenting/assenting, interviews with the child, questionnaires with parents, and the FITs. Assessors brought all the necessary materials with them, including a device with a mobile hotspot, video recorder, physical copies of the questionnaires, interview and observational protocols, and materials needed for the FITs (i.e., the game board required for the FITs task measuring parents’ encouragement of their child). Staff would read the families the instructions for each of the FITs and then leave the room to facilitate privacy and attempt to prevent other family members from entering the room. Tasks in which both parents were participating were conducted in both dyad and triad pairs (i.e., mother–child, father-child, mother-father-child) and in single-parent families or families in which only one parent was participating, only dyadic tasks were conducted (i.e., mother–child or father-child). A comprehensive description of the in-home study procedures can be found in Gewirtz et al., (2014, 2018), and a sample of the virtual manual given to assessment technicians can be found in the Supplementary Materials.

### Observational Data Collection

A total of *n* = 39 assessment technicians were trained for the SMART study. Each assessment technician received 14 h of initial training, including training in observational data collection and methodology and identifying circumstances that would warrant mandated reporting. This training culminated in a mock assessment conducted with senior staff as an opportunity to receive additional practice, coaching, and feedback. For context, the original in-person observational data were video recorded by the assessment technicians on a password-protected study device (iPad) using a password-secured software system with access restricted to relevant study staff. Recordings were temporarily stored in the secured software system until the assessment technicians took the device to the site supervisor who would upload the video to the secured online portal for permanent storage and safely store the device. Observational coders who were blinded to each family’s randomization condition accessed the recordings from this secured online portal.

The COVID pandemic and subsequent lockdown resulted in a mandated halt of all research activity from March to December 2020. This pause resulted from a combination of university regulations, restrictions in each state data were collected, the uncertainty in how long restrictions would be in place, and the logistic and methodological challenges associated with transitioning to fully remote data collection. Because of the rolling nature of recruitment and because of the later addition of the FBM site (with recruitment beginning only in 2019), prior to the lockdown, *n* = 186 baseline and *n* = 21 1-year follow-up assessments had been collected. In December 2020, studies were allowed to continue but only in an online or remote capacity. As such, the peri-COVID period presented a natural opportunity to develop and evaluate fully remote observational data collection during the remainder of the study. To achieve this, significant changes to the protocol and study methodology were required.

#### Transition to Fully Remote Data Collection

Over the 9-month period that research activities with participants were halted, the study team worked to modify the study protocol and procedures while retaining the integrity of the FITs. They began by brainstorming what ethical, methodologically sound, virtual observational data collection might look like. This included identifying which portions of the in-home assessments would require modification (e.g., addressing confidentiality or calling parents over the phone while their child participated on Zoom), consulting with the coding lab manager about potential challenges to conducting the FITs, and best practices to preserve data collection and quality. In this process, the study team drafted a new protocol for assessment technicians to follow. This draft was iteratively tested and refined by practicing with multiple staff members and then finalized. Changes to the protocol were submitted and accepted by the university IRB.

#### Fully Remote Data Collection

After research activities were allowed to resume, *n* = 7 of the original 39 assessment technicians received an additional 5–8 h of training in fully remote observational data collection. Their additional training focused primarily on the use of teleconferencing platforms (i.e., Zoom) and facilitating successful data collection without the ability to be in the home with the families (e.g., managing confidentiality, how to respond if the family is not in the video frame, or what to do if the family experienced internet connectivity issues). Assessment technicians were also coached on how to build rapport with families in a virtual environment, which often required them to act more energetically in order to create a similarly positive dynamic to being in the home. During this time, assessment technicians continued to test and refine the new assessment protocol.

As data collection resumed, parents were provided a Zoom link and password, a Zoom user tip sheet, and headphones to aid in confidentiality. Parents were instructed to set up their Zoom cameras in a private space, where possible. Children’s assent was obtained virtually in the presence of both the parent and the child, and families were coached to maintain privacy during the recording (i.e., to not allow other family members into the room), although the potential of other family members appearing on video was addressed in their informed consent. Required physical materials (e.g., the game board required for one of the FIT tasks, headphones as needed) were mailed to families in advance of their scheduled assessment. Parents were also provided with a document of available mental health resources at the same time staff sent them the confirmation of their virtual assessment appointment. During the FITs, after reading instructions to the parent/child pair, assessment technicians muted their microphones and turned off their cameras to replicate the privacy that was achieved by leaving the room when they were in-person.

The portion of the assessment in which FITs were conducted was recorded to a university-affiliated study Zoom account, rather than to the Zoom account of each individual assessment technician. Then, recordings were downloaded from Zoom and subsequently uploaded to the university’s academic health center’s HIPAA-compliant server. From there, the recordings were uploaded to the same secure study portal used for in-person data collection, confirmed, and then immediately deleted from the study Zoom account. During all downloading and uploading, study staff were connected to a virtual private network and could only access recordings with password-protected accounts. A total of *n* = 103 baseline, *n* = 166 of the 1-year follow-up, and all *n* = 89 of the 2-year follow-up assessments were collected in a fully remote capacity.

### Observational Data Coding

A total of *n* = 37 coders were trained over the course of the study, with a total of *n* = 27 coders from four cohorts coding baseline data (two cohorts coding in-person videos and two cohorts coding remotely collected videos), and a total of *n* = 10 coders from two cohorts coding the 1- and 2-year post-baseline data, taking care that no coders coded the same family twice. New cohorts of coders were recruited and trained at various points in time across the study, as they largely consisted of advanced/upper-year undergraduate students. All coders received 20 + h of initial training in the FITs coding manual, observational data methodology, coding observational data and reliability, maintaining participant confidentiality, and viewing and rating micro-expressions (brief facial expressions that can provide information about a person’s emotional state). Coders then completed another 20–30 h of practice training on pre-selected reliability training videos that had been pre-rated by a reliability coder (the coding lab manager). During this period, coders met for weekly reliability meetings in which video segments from that week’s assignment were reviewed as a group, followed by co-coding and intensive feedback facilitated by the coding lab manager.

Once the cohort achieved and sustained good to excellent reliability (measured by achieving an intraclass correlation coefficient (ICC) of > 0.60 on all tasks) with training videos for 2 or 3 weeks, they were assigned both reliability and individual videos. Coders were typically assigned two reliability videos per weekly or bi-weekly meeting, in addition to two or three individually coded videos. Coders participated in weekly or bi-weekly reliability and recalibration meetings to check reliability and minimize inter-rater drift. These meetings were conducted in a similar manner to those conducted during their training period.

#### Transition to Fully Remote Coding

Since all research activities were suspended from March to December 2020, coding also ceased while videos were not being collected. The study team and coding lab manager began work to inform the protocol for the assessment technicians by piloting different devices that families could use while participating in remote FITs. It was determined that laptops and desktops with cameras were preferable, but that tablets could be used if the family did not have either. However, cell phones were not usable, as the camera lenses were not wide enough to capture all family members on screen. This testing revealed difficulties in achieving the correct angles required to see both the family members and the game board used during the encouragement FIT. For this task only, assessment technicians were instructed to have families angle their cameras to see the game board, pieces, and participants’ hands. This choice was made because the coding manual necessitated seeing the game board and participants’ hands, but not necessarily their faces. No changes were made to the coding manual or coding protocol. The first few remotely collected FITs were immediately coded to ensure there were no additional unanticipated issues, at which point the video and audio quality were determined to be adequate for coding.

#### Fully Remote Coding

In December 2020, coders were retrained, both to complete the coding of the in-home videos already gathered and to prepare to code the COVID-protocol (i.e., Zoom) videos. Pre-COVID, as standard practice, coders were asked to add comments on their coder data regarding any concerns about the quality of the data gathered as it pertained to the ability to code. Peri-COVID, these notes focused on any challenges of coding data via Zoom (e.g., placement of microphone that may have affected audio quality and background noise).

In addition, coders were trained, and reliability meetings were held remotely via a teleconferencing platform. In an attempt to minimize “Zoom fatigue” common during lockdown, coder training was divided over the course of several days. Coders were required to keep their video cameras on during training and reliability meetings to help trainers and the coding manager verify attention and comprehension. To maintain participant confidentiality both during training and while coding study data, coders were required to attend meetings with the coding team from a private location (i.e., in a room by themselves using headphones). Pre-COVID, coders had access to a private coding lab equipped with secure computers, through which they accessed videos and submitted their ratings if needed. However, even pre-COVID, coders primarily worked remotely. Onboarding for all coders included ensuring that each individual had a laptop/tablet, access to stable, secure internet (i.e., not just public-use internet), and a private workspace where they could code videos uninterrupted and without risk of compromising privacy. While coding, coders were required to set up a university-supported virtual private network, after which they would access the secure database storing participant videos.

## Measures

### Feasibility

The feasibility of observational data collection both pre-COVID and after research activities resumed was measured by examining coder impressions written by coders as they rated each video. The trained coders assessed audio and video quality while observing and rating each video recording of the FITs collected both in families’ homes and via teleconferencing. Coders noted factors associated with whether they could see and hear well enough to code the video accurately and reliably. Technical challenges were rated on a 4-point Likert-style scale where 1 indicated “*nearly impossible to code some tasks or sections*” and 4 indicated “*no noticeable problems*.” If coders experienced difficulties in a particular video, they then provided free-text responses describing that challenge. Examples include “*there was a baby crying in the background that made the parent hard to hear during X task*,” “*child spoke too quietly to understand during X task*,” and “*father spoke in Spanish periodically throughout the video*.” Coders also rated whether assessment administration made it hard to code (e.g., missing or out-of-order tasks, incorrect instructions given, too long or too short time given for tasks). Administration problems were rated on a 4-point Likert-type scale where 1 indicated “*major administration problems—nearly impossible to code some tasks or sections*” and 4 “*no noticeable administration problems*.”

### Reliability

Reliability for observational data collected both pre- and peri-COVID was measured via the use of ICCs calculated with coder’s ratings for randomly selected reliability videos. A total of *n* = 207 videos were recorded in-person (186 at baseline and 21 at 1 year) and *n* = 358 were recorded virtually peri-COVID (103 at baseline, 166 at 1 year, 89 at 2 years post-baseline). A subset of FIT videos was rated by six cohorts of coders for inter-rater reliability. Three groups of coders (*n* = 6, 4, and 6 coders, respectively) rated the in-person recorded videos (with 11, 13, and 16 videos, respectively) and another set of three groups of coders (*n* = 5, 4, and 3 coders, respectively) rated the virtually recorded videos (with 14, 16, and 26 videos, respectively).

### Covariates

Demographic information was collected from each family. This included, but was not limited, to child age (in years), parent age (the average of both parents, in years), parent education level (the average of both parents, 1 = *High school or less*, 2 = *some college*, 3 = *associate*, 4 = *bachelor’s*, 5 = *graduate level*), and family income (1 = *less than $50* k, 3 = *$50 to $100 k*, 5 = *$101–$150 k*, 7 = *more than $151 k*).

## Data Analysis

### Feasibility

Challenges described by coders were categorized into common themes by three authors (QC, ST, and SL). A general inductive approach was used to identify emergent themes (Thomas, 2006). Two teams from the three authors (QC-SL and ST-SL) read each comment, discussed, and categorized them into five themes: audio problems, visual problems, logistical/administrative problems, distraction, and internet problems. A hybrid of consensus coding and split coding was used to classify themes. One-third of the comments were classified using consensus coding between two authors, and the rest were classified using split coding where authors divided the comments, coded them separately, and resolved issues and inconsistencies through discussion. Logistic and linear regressions were used to examine whether there were significant differences in the frequency and severity of each type of problem, respectively, before and during the COVID period, controlling for child age, parent age, parent education level, and family income.

### Reliability

To examine coder inter-rater reliability, intraclass correlation coefficients (ICCs) were calculated. The purpose of estimating inter-rater reliability was to assess consistency in the mean ratings, instead of absolute agreement, between multiple coders. Unlike kappa statistics, which estimate the degree of consensus between two (or more) raters after correcting for agreement by chance, ICC is useful in calculating the degree of consistency between raters when the data are continuous. Participants and coders were considered random samples from larger populations. Therefore, two-way random effects models were applied to calculate the ICCs, and average-measure ICCs were used in this study. ICC values less than 0.40 are indicative of poor inter-rater reliability, values between 0.40 and 0.59 indicate fair reliability, values between 0.60 and 0.74 indicate good reliability, and values between 0.75 and 1.0 indicate excellent reliability (Hallgren, 2012).

**Fig. 1:**
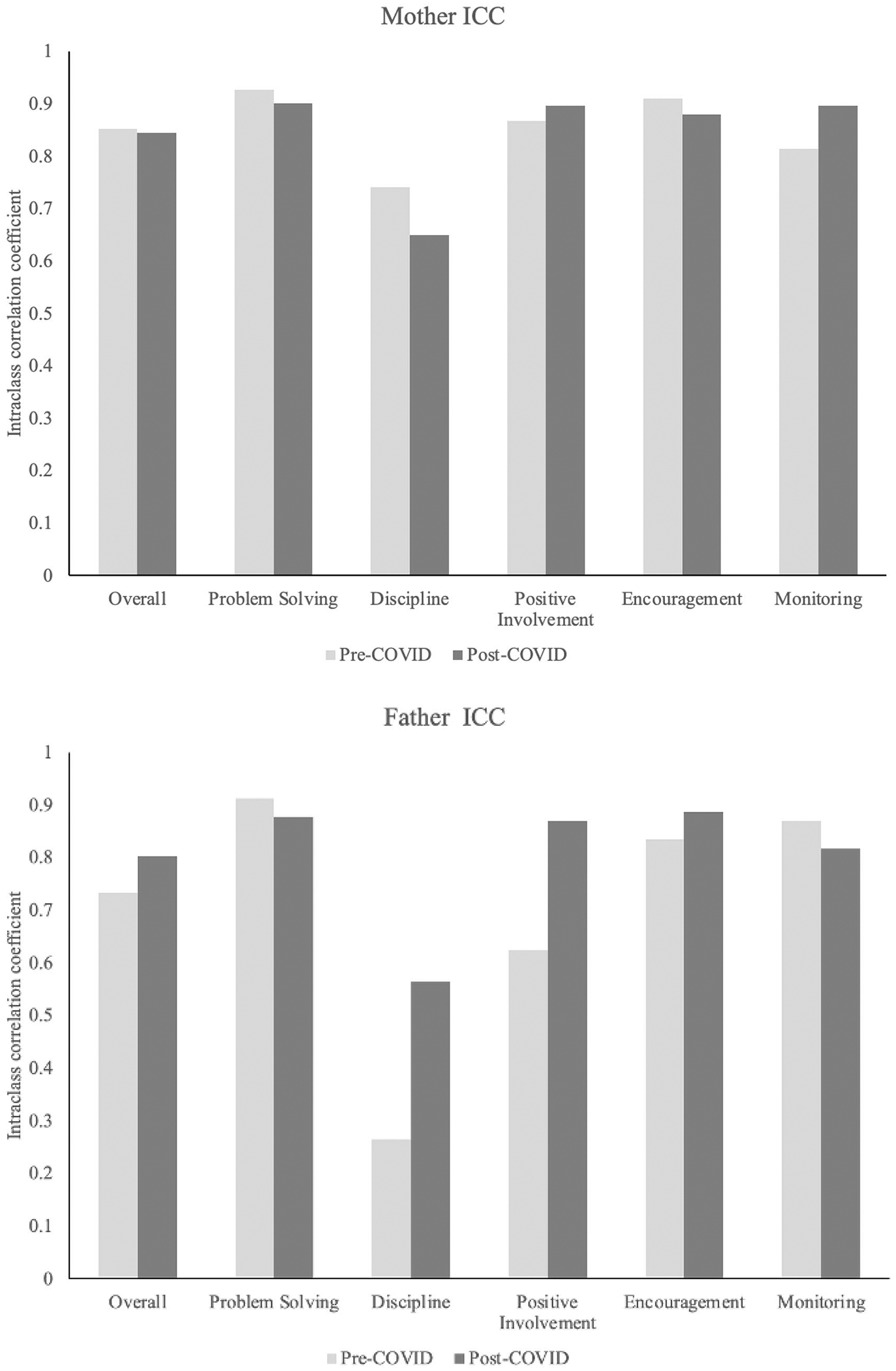
Inter-rater reliability for mothers and fathers pre- and post-COVID

Pre- and peri-COVID ICCs of the five measured domains of parenting skill (problem-solving, discipline, positive involvement, encouragement, and monitoring; shown in Fig. 1) were each calculated by taking the mean of the three ICC values from the in-person videos and then taking the mean ICC values from the virtually recorded videos. In previous research, these measures of parenting skill have demonstrated good to excellent reliability (problem-solving [0.85–0.91], discipline [0.59–0.88], positive involvement [0.76–0.89], encouragement [0.72–0.78], and monitoring [0.66–0.86]; Gewirtz et al., 2024). More detailed information about the parenting skills measured and intervention effects on parenting skills can be found from prior trials of the ADAPT program in Gewirtz et al., (2018, 2024).

## Results

### Feasibility

A total of *n* = 207 videos were recorded in-person pre-COVID, and *n* = 358 videos were recorded virtually peri-COVID across three time points. Table 2 summarizes coders’ impressions on challenges related to observational data collection in this study, and Table 3 summarizes the results of the regression analyses.

Coders reported encountering audio-related challenges in about a third of all videos, including quiet voice, background noise, and mumbling. Significantly more coders mentioned audio-related difficulties for the in-person videos (44.9%) compared to the virtually recorded videos (26%; *β* = − 0.77, SE = 0.17, *p* < 0.001, OR = 0.46). Coders reported encountering visual challenges in about 10% of all videos, such as video angles not adequately capturing participants’ facial expressions, participants moving away from the camera, the camera being too far away to see facial expressions well, dim lights, etc. The frequencies of visual problems were not significantly different between in-person videos (13%) and remotely recorded videos (9.8%; *β* = − 0.27, SE = 0.28, *p* = 0.34, OR = 0.77).

Slightly more logistical problems, although non-significant after controlling for demographics, were reported for pre-COVID (7.7%) than peri-COVID videos (3.4%; *β* = − 0.74, SE = 0.4, *p* = 0.06, OR = 0.47). Examples included missing tasks/instructions or tasks being administered in the wrong order. Significantly more distraction-related problems were reported peri-COVID (7.5%) than pre-COVID (2.4%; *β* = 1.18, SE = 0.5, *p* = 0.02, OR = 3.27), such as the child leaving the room during tasks and other family members (e.g., siblings) interfering with parents’ ability to finish the task. A small proportion of the videos were impacted by internet problems (3.1%) peri-COVID, and no internet problems were reported for in-person videos, thus no logistic regression was conducted analyzing internet problems.

Table 4 presents the means and standard deviations of coders’ ratings on the three quality questions, and Table 5 presents the linear regression results. On average, coders reported low difficulties with coding related to audio quality for both in-person (*M* = 3.90, *SD* = 0.29) and remotely recorded videos (*M* = 3.90, *SD* = 0.30; *β* = − 0.004, SE = 0.03, *p* = 0.88). Coders reported significantly less severe difficulties with visual-related problems peri-COVID (*M* = 3.66, *SD* = 0.57) compared to pre-COVID (*M* = 3.44, *SD* = 0.70; *β* = 0.2, SE = 0.05, *p* < 0.001). In addition, coders rated significantly lower levels of administration problems for coding remotely recorded videos (*M* = 3.97, *SD* = 0.23) compared to pre-COVID videos (*M* = 3.90, *SD* = 0.37; *β* = 0.07, SE = 0.03, *p* = 0.01).

### Reliability

Figure 1 depicts the mean ICC values of pre- and peri-COVID ratings of mothers’ and fathers’ parenting behaviors. The ICCs show that the coding team was able to maintain good to excellent inter-rater reliability from pre- to peri-COVID for problem-solving, encouragement, and monitoring for both parents and positive involvement for mothers. Positive involvement of fathers showed improved inter-rater reliability for the peri-COVID recorded videos compared with the pre-COVID videos. The ICCs for discipline, however, were overall low, especially when coding the father’s discipline behaviors. Figure 1 shows that the inter-rater reliability of coded mother’s discipline behaviors declined peri-COVID, while ICCs of coding father’s discipline behaviors showed some improvement.

## Discussion

This is one of the first studies to describe and examine the feasibility and reliability of transitioning an established parent–child observational protocol from an in-home to a fully online/remote setting. The FITs used in this study have been shown to be reliable and valid in-person measures over decades of parenting intervention research (e.g., Forgatch & DeGarmo, 1999; Gewirtz et al., 2018). Although there are challenges for virtual approaches to observational data collection, this study demonstrated that observational data can be feasibly and reliably collected via fully remote methods.

Somewhat surprisingly, coder comments indicated *fewer* barriers to high-quality coding during the COVID pandemic when observational tasks were delivered remotely. Specifically, the frequency of audio, visual, and logistical problems was lower peri-COVID during remote data collection than pre-COVID during the in-home assessment collection, and audio problems were significantly lower. Distraction-related problems were reported to be significantly greater peri-COVID than pre-COVID. Not surprisingly, internet connection difficulties were present peri-COVID since an internet connection was not required for pre-COVID observational data collection, though at 3.1% of videos, the incidence of internet connection problems was very low (just *n* = 11 videos). Interestingly, while audio problems were less frequent during remote data collection, their severity was similar to audio problems noted for observations gathered via in-home assessment, suggesting that the same kind/severity of problems (quiet voice, background noise, mumbling) were evident peri-COVID. Despite assessment technician efforts, these are issues that can be difficult to correct (e.g., a participant with a quiet voice may find it hard to speak louder for the entire assessment).

When coders were asked to rate the degree of difficulty in hearing or seeing and the severity of administration problems, coders reported higher levels of difficulties in seeing well enough to code and higher levels of administration problems for in-person pre-COVID videos. With regard to audio/visual challenges noted by coders, it is likely that videos collected in-person were somewhat subject to the experience of the assessment technicians. As the in-person videos were collected prior to the remote videos, they were naturally collected when the assessment technicians were newer, and while the study staff were addressing audio/visual challenges as they occurred. Regarding administration challenges, it is possible that, for in-home assessments, data collection staff had more distractions and tasks to manage than when they were remote. For example, during remote data collection, it is not staff but parents who are responsible for managing the set-up of the room, the placement of the camera, and managing other children or family members in the household, with staff primarily conducting quality control. Of note, logistical problems, including missing tasks or instructions, or tasks being administered in the wrong order, were far more prevalent pre-COVID. It seems that when assessment technicians are not physically present with families, they are able to follow their protocols more precisely. Indeed, it is far easier to read the manual when it is on the same screen as the participants than when reading it or referring to it when it “stands” physically between the technician and participants.

Both with in-home and remote data collection, study coordinators conducted brief calls with parents to schedule and prepare them for the assessment by asking them to ensure they would have privacy, sufficient space to conduct the assessment (i.e., at least two rooms so that parent and child could be interviewed separately, a table to place the games on, and chairs for the triad or dyad). During peri-COVID data collection, the focus of the call was also to ensure that parents had stable internet, would be able to manage the different tasks, and knew to expect the materials in the mail.

Due to the remote nature of data collection, the study team was able to manage with fewer assessment technicians peri-COVID than pre-COVID, and it is likely that the expertise of those technicians was greater as they had more practice and more experience delivering assessments. The advantage of a more highly skilled and smaller team was evident in closer, more frequent, and formal and informal communication with the assessment manager. Far less ongoing training was required, and turnover was less, which additionally saved on human resources costs. This study did not gather cost data here, but it is anecdotally clear that costs were far lower for remote than in-person data collection. While added costs for remote data collection included mailing packages to families and providing the game board needed for the FITs encouragement task (which was left with families as a gift after the observations were completed), these costs of approximately $40 per family were significantly lower compared to the cost of the personnel travel time and mile-age reimbursement associated with conducting in-person assessments (which varied widely from a minimum of $100 to several hundred dollars).

Reliability of the observational coding was similarly improved for pre- vs. peri-COVID era video recordings for both mothers and fathers on four of the five parenting dimensions. ICCs for all mother parenting domains, except for discipline, exceeded 0.80 across pre- and peri-COVID coding. For father domains, all ICCs except in the discipline domain were 0.80 or higher for remote, but not in-person data collection. In general, and throughout previous ADAPT studies, research has found father’s parenting practices to be harder to code than mothers. One potential explanation for this finding is that pre-COVID, mothers tended to take a more prominent role in the triadic FITs (i.e., those with mother-father-child) than fathers.

However, while the reliability of coding for mother’s discipline in these videos decreased for remote data collection, the coding reliability of father’s discipline improved from in-home to remote data collection. It is unclear why this is, but one potential explanation is derived from the fact that, in the majority of the families in the sample, fathers were the deployed parent. Because fully remote data collection occurred during lockdown, most families were required to stay home together potentially causing shifts in co-parenting roles. Additionally, in general, there was an overall low incidence of discipline issues in this sample of children which resulted in difficulties observing parents’ discipline behaviors and many data points with zeros (*never*). In the future, selecting tasks that pull for more discipline behaviors (e.g., a task such as having parents instruct their child to clean up toys) might be more effective at yielding a more complete assessment of discipline behaviors.

In sum, feasibility and inter-rater reliability data suggest no decrements in either the quality of observational data or its coding resulting from remote vs. in-person assessments, with some tangible benefits to remote data collection. While the study team is unlikely to revert to in-home assessments given the feasibility, reliability, and lowered costs of remote observational data capture, it should be acknowledged that there may be some drawbacks to remote assessments that were not fully captured in this study. For example, assessors in the home are able to absorb context about the family (e.g., state/organization of the home, presence of other family members and friends, quality of the neighborhood) that remote data collection cannot capture. Privacy concerns, such as individuals sitting unseen to the camera and observing or listening to what the respondent is saying (whether adult or child), are important, and there are limitations to the degree to which these can be resolved.

In addition, the sample for this study was primarily White and concentrated in three geographic areas (NC, KY, VA), although these included both rural and urban neighborhoods. This sample overwhelmingly had high-speed internet access, likely associated with their proximity to military installations; however many families, particularly low-income families in rural areas, do not have reliable high-speed internet access. Another important, albeit unavoidable, limitation relates to the coding team. Although significant care was taken to ensure that each cohort of coders was trained and maintained reliability (with consistent reliability indeed achieved across cohorts), the possibility that individual person or cohort differences did not contribute to the variability in outcomes for this study cannot be fully eliminated. Future research should explore individual differences that may contribute to variance in observational coding. Finally, despite its drawbacks, this study is one of the first to compare both the feasibility and reliability of parent–child observations from the in-home-to-remote transition. The presented data suggest that not only are remotely gathered observations feasible, but also that the coding of these observations is reliable, and these data should provide a degree of optimism to those who value the importance of observational data collection as a key method to assess parenting practices.

## Supplementary Material

Sup Materials

## Figures and Tables

**Table 1:** Parent, child, and family demographics, split by those assessed pre- and peri-COVID

Individual demographic	Pre-COVID (*n* = 305 adults)	Peri-COVID (*n* = 161 adults)	*t* or χ^2^	*p*
Parent age	34.42 (4.90)	37.55 (5.53)	−6.27	< .001
Parent education level			24.84	< .001
High school or less	31 (10.2%)	11 (6.9%)		
Some college	97 (31.8%)	41 (25.8%)		
Associate’s	43 (14.1%)	17 (10.7%)		
Bachelor’s	100 (32.8%)	43 (27.0%)		
Graduate level	34 (11.1%)	47 (29.6%)		
Parent gender—male	126 (41.3%)	63 (39.1%)	0.21	.648
Parent race			1.35	.509
African American	28 (9.4%)	11 (7.0%)		
White	239 (80.5%)	126 (80.3%)		
Other	30 (10.1%)	20 (12.7%)		
Parent marital status			5.11	.078
Married	285 (93.4%)	158 (98.1%)		
Divorced/separated	18 (5.9%)	3 (1.9%)		
Never married	2 (0.7%)	0 (0.0%)		
#Deployments	3.48 (2.94) *n* = 157	3.18 (2.34) *n* = 76	0.78	.438
Family or child demographic	Pre-COVID (*n* = 187 families)	Peri-COVID (*n* = 102 families)	*t* or χ^2^	*p*
Household income			28.69	< .001
Less than $50 k	56 (30.1%)	17 (17.0%)		
$51–$100 k	101 (54.3%)	42 (42.0%)		
$101–$150 k	23 (12.4%)	22 (22.0%)		
> $151 k	6 (3.2%)	19 (19.0%)		
Child age	7.97 (2.12)	8.67 (2.13)	−2.69	.008
Child gender–male	90 (48.1%)	49 (48.0%)	0.00	.988

**Table 2:** Frequencies of themes emerged from coders’ comments in coding in-person vs virtually recorded videos

Themes	In-person videos (*n* = 207)	Virtual videos (*n* = 358)	Chi-square
Audio problems	93 (44.9%)	93 (26%)	20.48**
Visual problems	27 (13%)	35 (9.8%)	1.12
Logistic problems	16 (7.7%)	12 (3.4%)	4.45*
Distractions	5 (2.4%)	27 (7.5%)	5.53*
Internet problems	0 (0%)	11 (3.1%)	/

* *p* < .05;

** *p* < .0001

**Table 3:** Logistic regression analysis of problem frequencies

	Audio problems			Video problems			Logistic problems			Distraction problems		
	beta (*SE*)	*p*	*OR*	beta (*SE*)	*p*	*OR*	beta (*SE*)	*p*	*OR*	beta (*SE*)	*p*	*OR*
Intercept	0.59; (0.7)	.40	1.80	−1.37; (1.03)	.18	0.25	−0.1; (1.47)	.95	0.90	−2.08; (1.44)	.15	0.13
Virtual (ref = in-person)	−0.77; (0.19)***	< .001	0.46	−0.27; (0.28)	.34	0.77	−0.74; (.4)	.06	0.47	1.18; (0.5)**	.02	3.27
Child age	0.07; (0.05)	.13	1.07	−0.03; (0.07)	.64	0.97	−0.12; (.11)	.27	0.89	−0.11; (0.1)	.26	0.89
Parent age	−0.03; (0.02)	.18	0.97	−0.02; (0.04)	.66	0.98	−0.01; (.05)	.81	0.99	−0.03; (0.05)	.53	0.97
Parent education	−0.02; (0.1)	.86	0.98	0.17; (0.15)	.25	1.19	−0.33; (.22)	.12	0.72	0.005; (0.21)	.98	1.00
Family income	−0.07; (0.07)	.31	0.93	−0.09; (0.1)	.35	0.91	−0.04; (.16)	.79	0.96	0.1; (0.14)	.45	1.11
Chi-square	28.24***			3.76			12.20*			9.48		
Hosmer–Lemeshow	12.53			1.17			7.99			11.2		
Hosmer–Lemeshow p	.13			.99			.43			.19		
Nagelkerke’s *R*^2^	.068			.01			.066			.048		

* *p* < .05;

** *p* < .001,

*** *p* < .0001

**Table 4:** Means and standard deviations of ratings on quality questions

	In-person videos *M* (*SD*)	Virtual videos *M* (*SD*)	*t*-test
Could you hear well enough to code?	3.90 (0.29)	3.90 (0.30)	0.11
Could you see well enough to code?	3.44 (0.70)	3.66 (0.57)	3.97**
Did assessment administration make it hard to code?	3.90 (0.37)	3.97 (0.23)	2.48*

Lower scores indicate greater difficulties

* *p* < .05;

** *p* < .0001

**Table 5:** Linear regression analysis of problem severity

	Audio problems		Video problems		Logistic problems	
	beta (*SE*)	*p*	beta (*SE*)	*p*	beta (*SE*)	*p*
Intercept	3.85; (0.09)	< .001	3.44; (.2)	< .001	3.83; (0.09)	< .001
Virtual (ref = in-person)	−0.004 (0.03)	.88	0.2; (.05)**	< .001	0.07; (0.03)**	.01
Child age	−0.01; (0.01)	.43	−0.02; (0.01)	.18	0.01; (0.01)	.28
Parent age	0.004 (0.003)	.23	0.001; (0.01)	.86	0.001; (0.003)	.87
Parent education	−0.01; (0.01)	.27	0.01; (0.03)	.69	−0.001; (0.01)	.93
Family income	0.003; (0.01)	.76	0.03; (0.02)	.14	0.002; (0.01)	.86
*R* ^2^	.004		.04		.017	

* *p* < .05;

** *p* < .0001

## Footnotes

^1^ For logistical reasons, however (i.e., the failure of a prior site), recruitment at FBM only began in 2019, 2 years into the study.
