# Supplementary material for: Does Being In-Person Matter? Demonstrating the Feasibility and Reliability of Fully Remote Observational Data Collection
Source: Prev Sci. 2024 Jul 12;27(1):16–27. doi: 10.1007/s11121-024-01706-6 (PMC11899375; doi:10.1007/s11121-024-01706-6)
Supplement: Supplementary file 1 — Supplementary file1 (PDF 948 KB) [file 11121_2024_1706_MOESM1_ESM.pdf]

Does being in the home matter? Feasibility and reliability of remote observational data collection in a randomized trial

Supplemental Materials

For more information or for questions, please contact:

Abigail H. Gewirtz, PhD

Foundation Professor

Department of Psychology

Arizona State University

abigail.gewirtz@asu.edu

Amy Majerle, MA

Senior Program Manager

Department of Psychology

Arizona State University

amy.majerle@asu.edu

These supplementary materials are a sample of the protocol/manual given to our technicians to conduct virtual assessments peri-COVID. **Part 1** includes protocol, instructions, and troubleshooting for the technicians prior to conducting the Family Interaction Tasks (FITs), including introductory information, confidentiality and informed consent, child assent, calling families, demographic and deployment verification, school release information, and setting up the family/providing instructions for the FITs. **Part 2** includes the step-by-step instructions, including scripts and troubleshooting, for conducting the FITs with a two-parent family.

ADAPT SMART STUDY

SMART VIRTUAL ASSESSMENT PROTOCOL: PART 1

PARENT TECH

PRIOR TO ASSESSMENT

Go to the family's folder to review the Family Information Form and General Demographic Verification Form for important information regarding the family (e.g. names, parent relationship to the target child, parent consent status, etc.).

MEETING THE FAMILY OVER ZOOM

The Zoom link used by the child and Child Tech during Part 1 will be sent to you in the scheduling email. At the time of the appointment, click the link to join the call. Be sure that both your video and audio are turned on.

WHAT TO DO IF THE FAMILY DOES NOT JOIN THE ZOOM CALL

If the family has not joined by 10 minutes after the scheduled assessment time, call the family (using the Google Voice instructions below) to see if they are having trouble connecting to the Zoom call, are no longer able to meet, etc.

Report any missed appointments to the site contact immediately.

Allow the Child Tech to introduce himself/herself to the child and parent. S/he will then give you an opportunity to introduce yourself.

Hi, I'm (_________). (Parent), I will be the one giving you a call in a little bit to walk you through some paperwork and to go over the videotaped portion that you'll be completing with (Child) later today. So I will say goodbye for now, and once (Child Tech) notifies me that you're done with this first part, I will give you a call. Nice to meet you, and I'll talk to you soon!

Click "Leave" in the lower right-hand corner of your screen to leave the meeting.

The child tech will text you to let you know once s/he has completed the assent process and that you may now call the parent.

CALLING THE PARENT(S)

1) Open a Google Chrome browser.
2) Go to voice.google.com
3) Make sure you are signed out of all Google accounts. If not, sign out and then return to voice.google.com
4) Click "Sign In" in the upper right-hand corner of the screen.

ADAPT SMART STUDY

5) Select "Use another account" in the "Choose an account" list.
6) Type in the email name (without "@gmail.com").
7) Type in the password.
8) If prompted to save the password, select "Never."
9) Use the keypad on the right-hand side of the screen to type the parent's phone number and hit the phone icon to dial.
10) A textbox will appear in the upper left-hand corner. Click "Allow" to complete the call.

If Google Voice's access to your microphone was blocked previously, you will need to change your browser settings.

1) In the Chrome browser, click the More icon (three vertical dots) in the upper right-hand corner.
2) In the dropdown menu, click "Settings."
3) Under "Privacy and security" on the left-hand side of the screen, click "Site Settings."
4) Underneath "Permissions," click "Microphone."
5) Google Voice will appear under "Block." Click the Delete icon next to it.
6) Return to Google Voice and redial the number. You should now be prompted to allow access to your computer's microphone.

Call phone number of (Mom/Dad). If it's a 2-parent family, the number that should be called will be established during the scheduling call and listed on the Family Information Form.

INTRODUCTION

Hello. This is _____________ calling from the ADAPT study. Is this (Parent's Name)? (If applicable), ***is (other parent) there with you as well? If you haven't already, could you put the phone on speaker so that you both can hear me?***

Spend a few minutes building rapport with them.

Do you have the envelope with the materials for today's interview with you? (If they do not, have them go get the envelope before continuing.)

If you open the envelope, the first piece of paper should say "ADAPT Interview Agenda" and gives a brief overview of today's activities. As you can see, while (Child) completes some survey questionnaires with (Child Tech), we'll work together to fill out some paperwork and pick topics for later discussions.

During the second part, we'll record you and (Child) having those discussions as well as doing a puzzle activity together.

Do you have any questions before we begin?

ADAPT SMART

INTRODUCTORY PAPERWORK

CONSENT FORM & CONFIDENTIALITY

Even though we strive to have each parent complete the consent form online prior to the first interview, some parents do not.

Check the Family Information form PRIOR TO THE INTERVIEW to verify that each participating parent has consented. If it indicates that one of them has not, verify with the site coordinator in case s/he consented after the paperwork was made.

If so, update the Family Info form. If not, follow the set of directions entitled "If one parent has NOT consented."

- If BOTH parents have consented

The next form in the envelope should say "Clinical Participant Consent Form" at the top. Do you see it?

This is the consent form you signed online prior to joining the study, so you don't need to sign it today. However, I do want to remind you that everything we talk about today is confidential except for a couple of situations.

We are required to report if either a) someone tells us about a child being harmed or b) if we think someone is in danger of harming themselves or someone else. Otherwise, everything we talk about today is considered confidential.

(If it's a 1-parent family): ***The copy is yours to keep.***

(If it's a 2-parent family): ***There should be two copies in there, one for each of you. They are yours to keep.***

- If one parent has NOT consented

The next form in the envelope should say "Clinical Participant Consent Form" at the top. Do you see it?

(To the parent who has consented): ***This is the consent form you signed online prior to joining the study, so you don't need to sign it today.***

(To the parent who has not consented): ***We also need a signed consent form from you before we can begin today. If you check your email, you should have just received a message with the ADAPT study consent form. Please click on the link, read through the form, and let me know if you have any questions.***
If after reviewing it, if you decide you want to participate in the study, you will sign the form electronically and submit it. I do not have access to the completed forms, so please let me know once you're finished.

YOU MUST VERIFY RECEIPT OF THE COMPLETED CONSENT FORM WITH THE SITE CONTACT BEFORE YOU ARE ALLOWED TO COLLECT ANY DATA FROM THE PARTICIPANT.

ADAPT SMART STUDY

Before moving forward, I want to remind you that everything we talk about today is confidential except for a couple of situations.

We are required to report if either a) someone tells us about a child being harmed or b) if we think someone is in danger of harming themselves or someone else. Otherwise, everything we talk about today is considered confidential.

The copy of the form is yours to keep.

ADAPT SMART STUDY

EXTRA ASSESSMENT FORMS

In the rare occurrence that we are only expecting one parent at the assessment, but the other parent decides to participate last-minute, you will find blank copies of any forms you may need here: [pathway name to restricted-access secure server]

CHILD ASSENT FORM

The next form you have should say "Assent" at the top. This is the form that (Child Interviewer) just read aloud to you and (Child) where you both gave your permission for (him/her) to participate today. It is simply a copy for your records.

FAMILY INFORMATION FORM

The next thing we need to do is briefly confirm your contact information.

Verbally verify the phone number and email address (for each parent, if applicable) as well as the family's physical address. (For example, ***(Parent), I have your phone number listed as xxx-xxx-xxxx. Is that correct?***)

BEFORE EDITING THE FORM, CHANGE THE FONT COLOR TO RED TO DIFFERENTIATE BETWEEN THE ORIGINAL CONTENT AND THE ADDITIONS/CORRECTIONS MADE BY THE ASSESSMENT STAFF.

- If the information is correct: click the box next to it to place an "x" in it.
- If the information is incorrect: click the strike-through button to cross out the incorrect information and type the correct information next to it.
- If the information is missing: gather the missing information from the parent and input it in the correct area.

Save the file and close out of it.

If the same email or phone number is listed for both parents, ask for different contact information for one of them.

MILITARY DEMOGRAPHIC VERIFICATION FORM

The next thing we need to do is make sure we have the correct information in regard to your military service.

Open the Military Demographic Verification Form in the family's folder. If it is a 2-parent family, ensure that you are on the first page and start with Dad.

Begin with Question 1 ("Have you ever served in the United States military?") and follow the flowchart to determine which additional questions, if any, need to be asked. The chart will be filled out as much as possible depending on the information the participant gave prior to the assessment.

Type an "X" in the box next to the correct response for each question.

If it is a 2-parent family, click on page 2 on the left-hand side of the screen and repeat the same process with Mom.

Save the file and close out of it.

T STUDY

FAMILY ELIGIBILITY

- The family's eligibility in the study is tied to their date of consent. In other words, one parent in the family (regardless of whether or not s/he is participating in the study) must have been Active Duty AND had a deployment in the last 5 years – or 2 deployments in the last 3 years in the case of SOF families – when they first consented to the study, even if that information is no longer accurate now.

- If a participant changes his/her response from "yes" to "no" for questions #3 or #5, rephrase the question, including the date of consent. (For example, ***Your family signed up to participate in the study in March of 2019. Had you been deployed overseas at least once in the five years prior to that date?***)
    - The date the parent(s) consented can be found on the Family Information form.

- Next Step:
    - If the participant responds "yes," place an "X" in the check box next to the response and continue with the assessment.
    - If the participant responds "no," ask for clarification (e.g. Have you been deployed overseas? When did you return from your last deployment?) and inform the family that you must contact the Site Coordinator for guidance before you can proceed.

We only gather demographic information for parents participating in the study. Therefore, if the parent who meets the eligibility criteria is not participating, there is no need for further follow-up.

GENERAL DEMOGRAPHIC VERIFICATION FORM

The next form in the envelope should say "General Demographic Verification Form" at the top. Do you see it?

I'll have you follow along on the form as I verify or gather some basic demographic information from you.

Verbally verify each of the items. (For example, ***Jane, I have your first name listed as J-A-N-E, and your last name as D-O-E. Is that correct?***)

BEFORE EDITING THE FORM, CHANGE THE FONT COLOR TO RED TO DIFFERENTIATE BETWEEN THE ORIGINAL CONTENT AND THE ADDITIONS/CORRECTIONS MADE BY THE ASSESSMENT STAFF.

- If the information is correct: click the box next to it to place an "x" in it.
- If the information is incorrect: click the strike-through button to cross out the incorrect information. Type in the correct information or click the correct check box, depending on the question.
- If the information is missing: gather the missing information from the parent and input it in the correct area.

IF THE CHILD DOB WAS CHANGED, NOTIFY THE CHILD TECHNICIAN IMMEDIATELY TO ENSURE THAT S/HE IS ADMINISTERING THE CORRECT ONLINE SURVEY.

If working with a 2-parent family

- Ask follow-up questions to resolve OR understand any discrepancies between the parents' responses for marital status, relationship status, or number of years in their current relationship.***
- Update responses as needed.

*** Different responses on these questions do not necessarily indicate error. For example, here are a few scenarios where a discrepancy would be appropriate:

1) Mom marks "divorced" and Dad marks "never married" for marital status
   a. This response would be appropriate if the couple is unmarried, but Mom is divorced from a previous partner (and Dad has never been married).

2) Mom marks "single" and Dad marks "in a relationship, living separately" for relationship status
   a. This response would be appropriate if the parents are participating in the study together (because they are co-parenting) but are no longer in a relationship with one another and Dad is now in a relationship with someone else.

Save the file and close out of it.

SMART STUDY

SCHOOL RELEASE FORM

We'd like to email your child's teacher and ask him/her to complete a short survey about (Child). The survey asks questions about (Child)'s behavior as well as (his/her) relationships with peers. We know this school year looks a lot different than other years and that teachers may be unable to respond to many of the items because of that, but teachers provide really valuable information, so we'd still like to be able to gather as much information from them as possible.

Would you be willing to let us contact (Child)'s teacher?

- If a parent consents

Okay, great. The next form in your packet should say "Release of Information Form" at the top and is what we will send to (Child)'s teacher, so that s/he knows we have your permission to send the survey. Please go ahead and fill in your name, (Child)'s name, and then sign and date the form.

Like the child assent, this form must be signed by a biological or adoptive parent. If we receive a form that is signed by a live-in partner or step-parent who has not adopted the child, we are unable to collect teacher data for that family.

- If a parent declines

Would you mind sharing why you wish to decline? We are asked to provide a brief explanation whenever we have missing data.

The parent's decline and explanation as to why should be recorded on the Parent Interviewer Impressions survey.

If a parent informs you that the child is homeschooled or is too young to attend school:

In that case, we would want to contact someone else who would be able to answer questions about (Child)'s relationships with peers. Ideally, this would be someone who has known (Child) for more than two months, sees (him/her) on an almost weekly basis, and is not a family friend or family member. Can you think of anyone who fits these criteria?

If parents need help coming up with ideas, you may offer age-appropriate suggestions, for example, a daycare provider, Sunday School teacher, soccer coach, etc.

Although the individual cannot be related to the child or be a family friend, it is not mandatory that s/he meet all three criteria. The idea is to find someone who knows the child well enough to provide reliable responses about his/her general behavior and peer interactions.

The alternative contact also cannot be someone the child has an expectation of privacy with, such as a therapist or counselor.

If an appropriate contact cannot be found, there is no need to have the parent sign the form. Make a note of it in the Parent Interviewer Impressions survey.

SCHOOL INFORMATION FORM

This form is only needs to be completed by parents who intend to give permission for us to contact their child's teacher or an appropriate alternative.

- If we'll be contacting a classroom teacher

On the back side of the form, it should say "School Information" at the top. This will be where you will provide information about (Child)'s school and (his/her) teacher.

I'll have you fill out the form now. Please let me know if you have any questions. Otherwise, just let me know when you are finished.

> If parents do not know the name of their child's teacher, or their child has multiple teachers and they don't know which one to list, they may ask the child for this information. In the case of multiple teachers, any teacher may be selected as long as s/he knows the child well.

The form can either be sent to the research team using the pre-addressed envelope in your packet, or you can take a picture of each side of it and email the pictures to the ADAPT email account. There should be a piece of paper paper-clipped to the envelope that has our email address. If you could return the form in the next day or two that would be fantastic. We oftentimes have a pretty short window to get information from teachers, so it's helpful to have the form as soon as possible.

- If we'll be contacting someone other than a classroom teacher

On the back side of the form, it should say "School Information" at the top. This will be where you will provide information about (Child)'s (daycare provider, etc.).

Please cross out the questions about (Child)'s school and grade. For question number three, write in the name of the individual we'll be contacting and his/her relationship to your child. And then please fill in his/her email address for question four.

Please let me know if you have any questions. Otherwise, just let me know when you are finished.

The form can either be sent to the research team using the pre-addressed envelope in your packet, or you can take a picture of each side of it and email the pictures to the ADAPT email account. There should be a piece of paper paper-clipped to the envelope that has our email address. If you could return the form in the next day or two that would be fantastic. We oftentimes have a pretty short window to get the information, so it's helpful to have the form as soon as possible.

If the parent indicates the intended method for returning the form, make a note of it on the Parent Interviewer Impressions survey.

TEACHER PERMISSION FORM

One thing that helps us gather information more quickly is when parents are able to let (teachers, daycare providers, etc.) know that we'll be contacting them.

ADAPT SMART STUDY

The next form in your packet should be a half sheet of paper. We will ask you to KEEP THIS FORM, fill it out, and give it to (Child)'s (teacher, daycare provider, etc.). If you don't have any in-person contact with him/her, we would still appreciate it if you could reach out through whatever means you typically communicate to relay the information.

PARENT CHECKLISTS

The checklists are used to generate topics for discussion during the family interaction tasks (FITs).

CHECKLIST 1: PROBLEM-SOLVING

It is essential that chosen topics be areas in which the parent and child disagree.

Next in the packet, is a form that says "PARENT CHECKLIST 1: PROBLEM-SOLVING" at the top. We are interested in how families talk about topics that may cause problems between parents and kids. So, later on today, you'll talk to (Child) about a topic you have chosen from this checklist.

I'll have you read through the following list and indicate how much of a problem each one has been between you and your child in the past two weeks. Let's do the first one together.

"Going to bed." Would you say that it has been a small, medium, or large problem between you and (Child) in the past two weeks?

If it's a 2-parent family, get responses from each parent before proceeding on, and make sure they know there is a checklist in the packet for each of them to complete.

If it's clear that they understand what to do, allow them to complete the checklist on their own. If they seem confused, go through the first few topics with them until you're sure they understand the instructions. Ask them to inform you when they've completed the checklist.

Open the Checklist 1 document in the family's folder.

- 1-parent family

After the parent has indicated s/he is finished, ***Can you tell me which topic numbers you marked as the largest problems?***

For each number the participant tells you, change the font color to red for both the topic number and name.

Pick one of the topics and ask them to briefly describe the problem. We ask for this information to ensure that the selected topic will make for a good problem-solving discussion (i.e. there is a great deal of disagreement between the parent and child). Continue asking about highly rated topics until you are confident you have selected a good one.

ADAPT SMART STUDY

Would you be willing to discuss (<u>chosen topic</u>) with (<u>Child</u>) later today?

If the parent declines to discuss it, continue going through his/her other highly rated topics until you find a good problem-solving topic that s/he agrees to discuss.

Type the number and description of the topic (for example, *#10 – Fighting with brother*) in the "Interviewer Only" box.

Save the file and close out of it.

- <u>2-parent family</u>

After the parents have indicated that they have finished, ensure you are on the first page of the checklist document and start with Dad.

Can you tell me which topic numbers you marked as the largest problems?
For each number the participant tells you, change the font color to red for both the topic number and name.

Repeat the process with Mom.

Starting with the parent who has the least number of highly rated topics, ask him/he to briefly describe the problem. We ask for this information to ensure that the selected topic will make for a good problem-solving discussion (i.e. there is a great deal of disagreement between the parent and child). Continue asking about highly rated topics until you are confident you have selected a good one.

Would you be willing to discuss (<u>chosen topic</u>) with (<u>Child</u>) later today?

If the parent declines to discuss it, continue going through his/her other highly rated topics until you find a good problem-solving topic that s/he agrees to discuss.

Type the number and description of the topic (for example, *#10 – Fighting with brother*) in the "Interviewer Only" box. Complete the same process with the other parent.

> <u>The topic chosen by one parent must be distinct from the topic chosen by the other.</u> When asking the second parent to describe the topic, make sure the description is different than that of the first parent's.
>
> For example, one parent may have indicated "using the television/electronics" was a large problem while the other indicated "how to spend free time" was. After inquiring further, you realize they are both talking about the same issue (the child spends all of his free time on his iPad).
>
> One parent may have this conversation with the child during the FITs, but a different topic must be selected for the other parent.

Save the file and close out of it.

CHECKLIST 2: DEPLOYMENT

It is essential that chosen topics be both emotional in nature as well as things that were difficult for the child.

Review the demographic verification forms to figure out who has deployed.

Open the Checklist 2 document in the family's folder.

Was (Child) old enough to remember the last time (you, your wife, his dad, etc.) was deployed overseas?

- If Yes

  Follow instructions for Scenario #1.

- If No

  Follow instructions for Scenario #2.

Parents who have been separated from their children due to deployment, training, etc. often express difficulty in completing this checklist because they obviously were not present to observe their child during that time. If this occurs, offer them the following:

We know this checklist can be difficult to fill out for the parents who were (deployed/separated), so just complete it as best you can. Think about conversations you may have had with (your spouse, the child's primary caregiver at the time, etc.) about any difficulties that (Child) experienced while you were away.

Scenario #1

Check the box next to "child remembers the deployment" at the bottom of the checklist.

IF BOTH PARENTS HAVE BEEN DEPLOYED, BUT THE CHILD WAS ONLY OLD ENOUGH TO REMEMBER ONE OF THEIR DEPLOYMENTS WELL, HAVE THEM FILL OUT THE CHECKLISTS IN THE CONTEXT OF THAT DEPLOYMENT.

We are also interested in seeing how families talk about topics related to deployment. So next in the packet, is a form that says "PARENT CHECKLIST 2: DEPLOYMENT" at the top. Later on today, you'll talk to (Child) about a topic that you have chosen from this checklist.

I'll have you read through the following list of deployment-related topics that your child may have encountered during a deployment and rate how hard each item was for (him/her). We'll do the the first one together.

ADAPT PART STUDY

"Bedtime routine without the deployed parent." Would you say that it was not at all hard, somewhat hard, hard, very hard, or extremely hard for (Child) during deployment?

If it's a 2-parent family, get responses from each parent before proceeding on, and make sure they know there is a checklist in the packet for each of them to complete.

If it's clear that they understand what to do, allow them to complete the checklist on their own. If they seem confused, go through the first few topics with them until you're sure they understand the instructions. Ask them to inform you when they've completed the checklist.

- 1-parent family

After the parent has indicated s/he is finished, ***Can you tell me which topic numbers you marked as hardest for your child during deployment?***
For each number the participant tells you, change the font color to red for both the topic number and name.

Pick one of the topics and ask them to briefly describe the problem. We ask for this information to ensure that the selected topic will make for a good problem-solving discussion (i.e. the topic is something that was difficult for the child and is emotional in nature). Continue asking about highly rated topics until you are confident you have selected a good one.

Would you be willing to discuss (chosen topic) with (Child) later today?

If the parent declines to discuss it, continue going through his/her other highly rated topics until you find a good problem-solving topic that s/he agrees to discuss.

Type the number and description of the topic (for example, *#14 – Seeing Mom sad or worried while Dad was deployed*) in the "Interviewer Only" box.

Save the file and close out of it.

- 2-parent family

After the parents have indicated that they have finished, ensure you are on the first page of the checklist document and start with Dad.

Can you tell me which topic numbers you marked as hardest for your child during deployment?

For each number the participant tells you, change the font color to red for both the topic number and name.

Repeat the process with Mom.

Starting with the parent who has the least number of highly rated topics, ask him/her to briefly describe the problem. We ask for this information to ensure that the selected topic will make for a good

ADAPT SMART STUDY

problem-solving discussion (i.e. the topic is something that was difficult for the child and is emotional in nature). Continue asking about highly rated topics until you are confident you have selected a good one.

Would you be willing to discuss (chosen topic) with (Child) later today?

If the parent declines to discuss it, continue going through his/her other highly rated topics until you find a good problem-solving topic that s/he agrees to discuss.

Type the number and description of the topic (for example, *#14 – Seeing Mom sad or worried while Dad was deployed*) in the "Interviewer Only" box.

Complete the same process with the other parent.

Save the file and close out of it.

Scenario #2

Check the box next to "child does not remember deployment" at the bottom of the checklist.

IF THE CHILD HAS EXPERIENCED MILITARY-RELATED SEPARATIONS WITH BOTH PARENTS BUT WAS ONLY OLD ENOUGH TO REMEMBER THE SEPARATIONS WITH ONE PARENT WELL, HAVE THEM FILL OUT THE CHECKLISTS IN REGARD TO THAT PARENT.

We are also interested in seeing how families talk about military-related separations. So next in the packet, is a form that says "PARENT CHECKLIST 2: DEPLOYMENT" at the top. Later on today, you'll talk to (Child) about a topic that you have chosen from this checklist.

I'll have you read through the following list of topics that your child may have encountered during these separations and rate how hard each item was for (him/her). We'll do the first one together.

"Bedtime routine." Would you say that it has been not at all hard, somewhat hard, hard, very hard, or extremely hard for (Child) during these separations?

If it's a 2-parent family, get responses from each parent before proceeding on, and make sure they know there is a checklist in the packet for each of them to complete.

If it's clear that they understand what to do, allow them to complete the checklist on their own. If they seem confused, go through the first few topics with them until you're sure they understand the instructions. Ask them to inform you when they've completed the checklist.

- 1-parent family

WHILE THE PARENT IS COMPLETING THE CHECKLIST, TEXT THE CHILD TECH TO LET HIM/HER KNOW YOU SHOULD BE FINISHED IN ~ 10 MINUTES AND GET AN ANTICIPATED END TIME FOR THE CHILD AND CHILD TECH.

After the parent has indicated s/he is finished, ***Can you tell me which topic numbers you marked as hardest for your child during military-related separations?***

ADAPT SMART STUDY

For each number the participant tells you, change the font color to red for both the topic number and name.

Pick one of the topics and ask them to briefly describe the problem. We ask for this information to ensure that the selected topic will make for a good problem-solving discussion (i.e. the topic is something that was difficult for the child and is emotional in nature). Continue asking about highly rated topics until you are confident you have selected a good one.

Would you be willing to discuss (chosen topic) with (Child) later today?

If the parent declines to discuss it, continue going through his/her other highly rated topics until you find a good problem-solving topic that s/he agrees to discuss.

Type the number and description of the topic (for example, *#14 – Seeing Mom sad or worried while Dad was deployed*) in the "Interviewer Only" box.

Save the file and close out of it.

- 2-parent family

After the parents have indicated that they have finished, ensure you are on the first page of the checklist document and start with Dad.

Can you tell me which topic numbers you marked as hardest for your child during military-related separations?

For each number the participant tells you, change the font color to red for both the topic number and name.

Repeat the process with Mom.

Starting with the parent who has the least number of highly rated topics, ask him/her to briefly describe the problem. We ask for this information to ensure that the selected topic will make for a good problem-solving discussion (i.e. the topic is something that was difficult for the child and is emotional in nature). Continue asking about highly rated topics until you are confident you have selected a good one.

Would you be willing to discuss (chosen topic) with (Child) later today?

If the parent declines to discuss it, continue going through his/her other highly rated topics until you find a good problem-solving topic that s/he agrees to discuss.

Type the number and description of the topic (for example, *#14 – Seeing Mom sad or worried while Dad was deployed*) in the "Interviewer Only" box.

Complete the same process with the other parent.

ADAPT SMART STUDY

Like Checklist 1, parents cannot choose the same topic.

Avoid overlap between checklists as well. For example, if a parent's topic for problem-solving (Checklist 1) is "fighting with brothers and sisters," do not choose the same topic for the deployment discussion (Checklist 2).

Save the file and close out of it.

CHECKLIST 3: CO-PARENTING

This checklist is for two-parent families only. It is not given to single-parent families or families where only one parent is participating in the interview.

It is essential that the chosen topic be one in which the parents disagree.

For example, for #1, "disciplining children," if both parents say that disciplining their children is difficult but that they generally agree on how to do it, we would not select this topic. Rather, parents would need to say that there is a great deal of disagreement between them on how their children should be disciplined.

Open the Checklist 3 document in the family's folder.

The final checklist in the packet should say, "PARENT CHECKLIST 3: CO-PARENTING. For this last one, we are interested in seeing how couples talk about topics related to parenting that may cause tension or conflict between partners. Later on today, you'll have the chance to talk with one another about a topic that relates to parenting in your family.

Please read the following list of topics that other couples have identified for us, and then rate how hard it has been to agree on your approach to each issue in the past two weeks. Once again, let's do the first one together.

"Disciplining children," would you say that it's been not hard, somewhat hard, hard, very hard, or extremely hard to agree on in the past two weeks?

Get responses from each parent before proceeding on, and make sure they know there is a checklist in the packet for each of them to complete.

If it's clear that they understand what to do, allow them to complete the checklist on their own. If they seem confused, go through the first few topics with them until you're sure they understand the instructions. Ask them to inform you when they've completed the checklist.

ADAPT SMART STUDY

WHILE THE PARENTS ARE COMPLETING THE CHECKLIST, TEXT THE CHILD TECH TO LET HIM/HER KNOW YOU SHOULD BE FINISHED IN ~ 10 MINUTES AND GET AN ANTICIPATED END TIME FOR THE CHILD AND CHILD TECH.

After the parents have indicated that they have finished, ensure you are on the first page of the checklist document and start with Dad.

Can you tell me which topic numbers you marked as the hardest to agree on?

For each number the participant tells you, change the font color to red for both the topic number and name.

Repeat the process with Mom.
Identify topics that were highly rated for both parents.

It sounds like (topic) is one of the more difficult topics to agree on. Can you tell me a little bit about that?

We ask for this information to ensure that the selected topic will make for a good discussion (i.e. the topic is something the parents have a difficult time agreeing on). Continue asking about highly rated topics until you are confident you have selected a good one.

Would you be willing to discuss (chosen topic) with one another later today?

If either parent declines to discuss it, continue going through other highly rated topics until you find a good problem-solving topic that they agree to discuss.

Type the number and description of the topic (for example, *#15 – How Jimmy uses his money*) in the "Interviewer Only" box.

Save the file and close out of it.

ADAPT SMART STUDY

TEACHING SHAPE-BY-SHAPE

Let's spend a few minutes talking about the game you'll be playing with (Child) later today.

In the packet you were mailed, there should be a game board with red and yellow puzzle pieces and an envelope that says "Shape-by-Shape Cards." Do you see all of that?

Once the parent confirms: ***I'd like you to open the envelope and pull the first paper-clipped section of cards out. If you remove the paper clip and look at all three cards, they should say "Demo #1 - Child," "Demo #1 - Parent 1," and "Demo #1 - Parent 2" at the top. Do you have those cards?***

Once the parent confirms: ***The goal of this game is to position the red and yellow pieces to match the design on the card that says "Child" at the top. You'll need to give (Child) the help you think (s/he) needs to rearrange the pieces to match the picture.***

That card is the only one we'll have you display to (<u>Child</u>), but you will the have key cards – the ones that say "Parent 1" and "Parent 2" at the top - that show how the individual pieces fit together to make the design.

If it's a 2-parent family: ***It doesn't matter which one of you has the Parent 1 card and which one has the Parent 2 card, as long as you each have a card.***

You may use the key cards to help instruct (<u>Child</u>). Do you have any questions about how the activity will work?

I also want to let you know that when you're playing the game later today, we'll be using a stopwatch to time the activity but only so the same amount of time can be given to each family. It's not a test to see how quickly you can complete the puzzle. And don't worry about remembering all of these instructions because we'll remind you of them later on.

PREPARING FOR THE FITS

The last thing I want to do is go over the set-up for the videotaped portion.

1) ***This part of the interview should be done seated at a table. Do you have a table and chairs you'd be able to use?***

If the family does not, so they have to sit on a sofa, the floor, etc., the explanation for this deviation in protocol should be noted on the Parent Interviewer Impressions survey.

2) ***To help make sure the video is able to capture your conversations and that there isn't too much background noise, we'll ask that anyone who is not participating in the activity not be in room if at all possible.***

3) ***In order to make sure you (<u>both/all</u>) are able to be seen on the video, __________, the (<u>Child Interviewer</u>) may ask you to make a few adjustments. For example, (<u>s/he</u>) may have you:***

    a. ***Reposition your chairs to be closer together or face more toward the camera***

    b. ***Turn on an overhead light if it's too dark or close blinds if it's too bright***

    c. ***Adjust the angle of your device or move it closer if it's not picking up sound well enough***

Do you have questions for me about the videotaping?

ADAPT SMART STUDY

TRANSITION TO PART 2

- If the child is still completing Part 1

If you have not yet heard from the child tech that they have completed Part 1:

You will now have a break until (Child Interviewer) and (Child) are done with the survey questions, probably about _________ minutes. (Give them an estimate based on your previous communication with the child tech.)

If you have time now, it would be great if you could either email the release of information form or get it ready to be mailed out. That way, you won't have to try to remember to do it later.

(Child Interviewer) will have (Child) come get you and let you know when they're ready for you. Otherwise, do you have any last questions for me?

Answer any last questions, thank the parent(s) for participating, and end the call.

- If the child has completed Part 1

If the child tech has informed you that they have completed Part 1:

(Child Interviewer) and (Child) are done with their first part of the interview as well. So now I'll have you and (Child) join the (Child Interviewer) on the Zoom call, but let (him/her) know if you need a break before starting Part 2.

And I know how busy parents are and how easy it is to forget, so I want to give you one last reminder to email or mail the release of information form in as soon as you can.

Otherwise, I will let you go unless you have any last questions for me?

Answer any last questions, thank the parent(s) for participating, and end the call.

ADAPT SMART STUDY

POST-ASSESSMENT

- Text the child tech to notify him/her that you have completed Part 1. Communicate any other important information (e.g. family said they do not have a table to sit at during FITs, you need five more minutes to finish updating the checklists, etc.)

- Review all of the forms in the family's folder to make sure you filled them out completely (including your interview ID number) and correctly.

- Sign out of the Google Voice account by clicking on the account icon in the upper right-hand corner of the screen and clicking "Sign Out."

- If applicable, communicate any time-sensitive information to the site coordinator.

- Complete the Parent Interviewer Impressions survey in Qualtrics. If working with a 2-parent family, complete a survey for each parent.

ADAPT SMART STUDY

SMART VIRTUAL ASSESSMENT PROTOCOL: PART 2

TWO-PARENT FAMILY INTERACTION TASKS (FITS)

If a Family ID ends in an even number, Dad and Child will complete the first task. If it ends in an odd number, Mom and Child will complete the first task.

ADAPT SMART STUDY

GENERAL OVERVIEW: EVEN-NUMBERED FAMILY

FAMILY INTERACTION TASK	PARTICIPANTS	TIME
1. Problem Solving 1 (Dad's Topic)	Dad, Child *(Mom – Break)*	5 min
2. Problem Solving 2 (Mom's Topic)	Mom, Child *(Dad – Break)*	5 min
3. Deployment Discussion 1 (Dad's Topic)	Dad, Child *(Mom - Break)*	4 min
4. Deployment Discussion 2 (Mom's Topic)	Mom, Child *(Dad – Break)*	4 min
5. Shape-by-Shape	Mom, Dad, Child	5 min
6. Co-Parenting Discussion	Mom, Dad *(Child – Break)*	5 min
7. Monitoring Discussion	Mom, Dad, Child	4 min
8. Fun Family Activity	Mom, Dad, Child	3 min
	Approximate Total	35 min

GENERAL OVERVIEW: ODD-NUMBERED FAMILY

FAMILY INTERACTION TASK	PARTICIPANTS	TIME
1. Problem Solving 1 (Mom's Topic)	Mom, Child *(Dad – Break)*	5 min
2. Problem Solving 2 (Dad's Topic)	Dad, Child *(Mom – Break)*	5 min
3. Deployment Discussion 1 (Mom's Topic)	Mom, Child *(Dad - Break)*	4 min
4. Deployment Discussion 2 (Dad's Topic)	Dad, Child *(Mom – Break)*	4 min
5. Shape-by-Shape	Mom, Dad, Child	5 min
6. Co-Parenting Discussion	Mom, Dad *(Child – Break)*	5 min
7. Monitoring Discussion	Mom, Dad, Child	4 min
8. Fun Family Activity	Mom, Dad, Child	3 min
	Approximate Total	35 min

TROUBLESHOOTING AUDIO/VIDEO ISSUES

Because it is imperative that we have good audio and video quality, you will continue to assess both throughout the FITs.

If participants are in the middle of a task and they experience connectivity issues:

VIDEO ONLY

- Allow the task to continue uninterrupted if we still have quality audio.
- Once the task is complete, troubleshoot the issues (e.g. having others in the home disconnect or asking the family to leave the session and rejoin).
    - If troubleshooting is successful, continue onto the next task.
    - If it is not (and you continue to have issues with the video), the FITs must be rescheduled.

AUDIO (regardless of whether or not the video is working)

- Stop the task if the audio is not working properly for A FULL MINUTE, making note of how much time had elapsed thus far.
- Troubleshoot the issues (e.g. having others in the home disconnect or asking the family to leave the session and rejoin).
    - If troubleshooting is successful, read the instructions to the task again and ask the family to resume where they left off. They will be given the remainder of the task time to discuss it. For example, if you stopped the task two minutes into a problem-solving discussion, you would give the family an additional three minutes after resuming.
    - If it is not (and you continue to have issues with either the audio or video), the FITs must be rescheduled.

GENERAL OVERVIEW

Are you ready to begin the videotaped portion?

Once they confirm (and if they are not already doing so), ask them to sit down at the table.

Before we begin the actual tasks, I'll give you some information about how things will work today.

I will let you know when I start recording the session. Once we begin, we will continue recording until you have completed the final discussion.

Some of the tasks will have all three of you present, and some will just have two of you. Whoever is not participating in a particular task will have about a 5-minute break during that time. The only thing we ask is that you take your break in a different area than where we're filming to help prevent distractions and problems with background noise.

During the tasks, I will give you instructions and then turn off my video while you complete your discussion. Once the time is up, I will turn my video back on and let you know that the task is complete.

Do you have any questions before we begin?

This first activity will be for (Child) and (Parent 1) so, (Parent 2), you have a short break now, and we'll have (Parent 1) come get you when it's time to return.

Parent 2 exits the room.

INITIATING THE RECORDING

I will begin recording now.

1. Click "Record" (near the bottom center of the screen).
2. Select "Record to the Cloud."
3. Verify the recording has begun by looking for the word "Recording" in the upper left-hand corner of the screen.

Before I give you instructions for the first task, I need to read a brief introduction.

This is ADAPT Family Interaction Tasks, Family ID (___) at Timepoint (___) on (date), with Interviewer (ID#).

Because you will not have the opportunity to stop the recording and play it to verify the accuracy of the header, it is essential that you take your time and make sure the header is recorded correctly.

Okay, we are ready to begin the first task.

Prepare your stopwatch - either one provided by study staff or the one on your phone.

ADAPT SMART STUDY

IF PARTICIPANTS ADDRESS YOU DURING A TASK

Because you are not physically leaving the discussion area (as you would during an in-person assessment), participants may be more likely to address you during a task. For example, they may let you know that they solved the issue or are done with their discussion.

If this were to occur, turn your video back off briefly and inform them: ***You still have a little bit of time left in this task. If you are done talking about the topic, you may talk about whatever you like, but please stay seated in these chairs and don't go on to talk about other topics you picked earlier. I will let you know when we've reached the end of the time period.***

Then turn your video off.

1: PROBLEM-SOLVING DISCUSSION - PARENT 1

Time: 5 min.

- Open Checklist 1 in the family's folder and scroll to Parent 1's checklist.
- Verify that the session is still recording.
- Check the screen to make sure the participants are properly framed. If they are not, make any needed adjustments.

We're interested in seeing how families talk about different types of topics. For the next five minutes, talk about one of the topics that you, (Parent), picked, which is (topic). Talk about what the problem is and try to figure out a way to solve it.

If you get done talking about this topic, you can talk about whatever you like, but please stay seated in these chairs, and don't go on to talk about the other topics you picked earlier. I'll come back in 5 minutes. Any questions?

Then you may begin.

- Start the timer.
- Click "Stop Video" and "Mute" on your Zoom screen.
- A few seconds before the five-minute mark, un-mute yourself and start your video.
- At EXACTLY five minutes: ***Okay, that is time.***

ADAPT SMART STUD

(Parent 1), the next discussion will be with (Child) and (Parent 2), so could you please let (Parent 2) know it's time to switch places?

2: PROBLEM-SOLVING DISCUSSION - PARENT 2

Time: 5 min.

- Scroll to Parent 2's checklist.
- Verify that the session is still recording.
- Once Parent 2 is seated and ready to begin, check the screen to make sure the participants are properly framed. If they are not, make any needed adjustments.

We're interested in seeing how families talk about different types of topics. For the next five minutes, talk about one of the topics that you, (Parent), picked, which is (topic). Talk about what the problem is and try to figure out a way to solve it.

If you get done talking about this topic, you can talk about whatever you like, but please stay seated in these chairs, and don't go on to talk about the other topics you picked earlier. I'll come back in 5 minutes. Any questions?

Then you may begin.

- Start the timer.
- Click "Stop Video" and "Mute" on your Zoom screen.
- Close Checklist 1.
- A few seconds before the five-minute mark, un-mute yourself and start your video.
- At EXACTLY five minutes: ***Okay, that is time.***

(Parent 2), the next discussion will once again be with (Child) and (Parent 1), so could you please go get (Parent 1)?

ADAPT SMART STUDY

3: DEPLOYMENT DISCUSSION – PARENT 1

Time: 4 min.

- Open Checklist 2 in the family's folder and scroll to Parent 1's checklist.
- Verify that the session is still recording.
- Once Parent 1 is seated and ready to begin, check the screen to make sure the participants are properly framed. If they are not, make any needed adjustments.

Part 1 of Deployment Discussion

✓ If the child remembers the deployment:

We're interested in families' experiences with deployment. (Parent) has picked a topic, and the two of you are going to talk about it for the next two minutes.

OR

✓ If the child does not remember the deployment:

We're interested in families' experiences when parents have to go away because of their work with the military. (Parent) has picked a topic, and the two of you are going to talk about it for the next two minutes.

For all families:

For the next 2 minutes, (Child), talk about (topic). Talk about what the problem was and how you felt when you were experiencing it. If you get done talking about the topic, you can talk about whatever you like, but please stay seated in these chairs.

I'll come back in 2 minutes with more directions. Any questions?

Then you may begin.

- Start the timer.
- Click "Stop Video" and "Mute" on your Zoom screen.
- A few seconds before the two-minute mark, un-mute yourself and start your video.
- At EXACTLY two minutes: ***Okay, that is time.***

ADAPT SMART STUDY

- Verify that the session is still recording.
- Check the screen to make sure the participants are properly framed. If they are not, make any needed adjustments.

Part 2 of Deployment Discussion

Now that you've talked about the topic and how you feel about it, it's time to think about what you would do if it occurred again. For the next two minutes, (Child), talk about how you might handle (topic) if it were to happen again.

I'll come back in 2 minutes. Any questions?

Then you may begin.

- Start the timer.
- Click "Stop Video" and "Mute" on your Zoom screen.
- A few seconds before the two-minute mark, un-mute yourself and start your video.
- At EXACTLY two minutes: ***Okay, that is time.***
- ***(Parent 1), I'm going to ask you to switch places with (Parent 2) one last time. Could you please let (him/her) know it's time to switch?***

4: DEPLOYMENT DISCUSSION – PARENT 2

Time: 4 min.

- Scroll to Parent 2's checklist.
- Verify that the session is still recording.
- Once Parent 2 is seated and ready to begin, check the screen to make sure the participants are properly framed. If they are not, make any needed adjustments.

Part 1 of Deployment Discussion

✓ If the child remembers the deployment:

We're interested in families' experiences with deployment. (Parent) has picked a topic, and the two of you are going to talk about it for the next two minutes.

ADAPT SMART STUDY

OR

✓ If the child does not remember the deployment:

We're interested in families' experiences when parents have to go away because of their work with the military. (Parent) has picked a topic, and the two of you are going to talk about it for the next two minutes.

For all families:

For the next 2 minutes, (Child), talk about (topic). Talk about what the problem was and how you felt when you were experiencing it. If you get done talking about the topic, you can talk about whatever you like, but please stay seated in these chairs.

I'll come back in 2 minutes with more directions. Any questions?

Then you may begin.

- Start the timer.
- Click "Stop Video" and "Mute" on your Zoom screen.
- A few seconds before the two-minute mark, un-mute yourself and start your video.
- At EXACTLY two minutes: ***Okay, that is time.***
- Verify that the session is still recording.
- Check the screen to make sure the participants are properly framed. If they are not, make any needed adjustments.

Part 2 of Deployment Discussion

Now that you've talked about the topic and how you feel about it, it's time to think about what you would do if it occurred again. For the next two minutes, (Child), talk about how you might handle (topic) if it were to happen again.

I'll come back in 2 minutes. Any questions?

Then you may begin.

- Start the timer.
- Click "Stop Video" and "Mute" on your Zoom screen.
- Close Checklist 2.
- A few seconds before the two-minute mark, un-mute yourself and start your video.

ADAPT SMART STUDY

- At EXACTLY two minutes: ***Okay, that is time.***

- ***This next activity is for all three of you so, (Parent 2), please let (Parent 1) know it's time to return. And when you sit back down at the table, we'll need (Child) to be seated between the two of you.***

5: SHAPE-BY-SHAPE

Time: 5 min.

- Verify that the session is still recording.

(Parents), we have some puzzles, for you to help (Child) do. Before we get started, I will have you go get the gray game board with the red and yellow pieces that we mailed to you as well as the cards that say "(Beginner/Intermediate/Advanced)" at the top.

Ages 5-8: Beginner
Ages 9-13: Intermediate
Ages 14 & up: Advanced

Once the parents have brought the needed materials to the table: ***Does the card on the top of the pile say "(Beginner/Intermediate/Advanced) #1 - Child"? And do the next two cards say "(B/I/A) #1 - Parent #1" and "(B/I/A) #1 - Parent #2"?***

Once the parents confirm: ***I'll have you take the child card and put it face up in front of (Child) and take the two parent cards and put one, face down, in front of each of you. I'll let you know when we're ready to turn them over.***

You should have six cards left in the pile, three with a #2 and three with a #3. Is that correct?

Once the parents confirm: ***I'll have you set those six cards, face down, off to the side, and I'll let you know if we need them later.***

Next, place the game board in front of (Child) and remove the pieces from the tray.

Check the screen to make sure you can see the table surface, including the game board, puzzle pieces, and participants' hands. If not, have them adjust the angle of the video EVEN IF THAT MEANS YOU CAN NO LONGER SEE THEIR FACES.

Okay, we should be all set up now, so I will go ahead and read you the instructions.

ADAPT SMART STUDY

The goal of the game is to put the yellow and red pieces in the right places to make the design shown on (Child)'s card.

(Child), your parents will have hints and can help you solve the puzzle if you need it. If you finish the puzzle within the 5-minute time period, remove the pieces from the tray, and begin completing the second puzzle. (Child), you should do as many as you can. (Parents), you should give (Child) the help you think (s/he) needs to solve the puzzles.

Do you have any questions?

And I also want to let you know that I will stay on video for this activity in case there are any additional instructions I need to give you as we go.

(Parents), you may now look at your cards and (Child), you may begin.

At EXACTLY five minutes: ***Okay, that is time.***

Nice job. It looks like you completed (#) card(s).

I will have you set aside the cards and puzzle pieces, and we will move on to the next task.

> IF THE FAMILY COMPLETES A PUZZLE WITHIN THE TIME FRAME
>
> ***Remove the pieces from the tray and, (Parents), grab the set of cards for puzzle #2. Give (Child) the one that says "Child" at the top, and make sure one of you has the "Parent 1" card and the other one has the "Parent 2" Card. You may go ahead and begin.***

(Child), this next task is just for your parents, so you can take about a 5-minute break. Your parents will let you know when it's time to come back into the room.

ADAPT SMART STUDY

6: CO-PARENTING DISCUSSION

Time: 5 min.

- Open Checklist 3 in the family's folder.
- Verify that the session is still recording.
- Check the screen to make sure the participants are properly framed. If they are not, make any needed adjustments. This may include asking a parent to move into the chair the child was sitting in previously.

This one's a little different than before. This time, we're interested in seeing how couples talk about topics related to parenting in their family.

For the next five minutes, please talk about the topic you agreed to discuss earlier, which is (topic). Talk about what the difficulty is and try to figure out a way to solve it. If you get done talking about it, you can talk about whatever you like, but please stay seated in these chairs. I'll be back in 5 minutes. Any questions?

Then you may begin.

- Start the timer.
- Click "Stop Video" and "Mute" on your Zoom screen.
- Close Checklist 3.
- A few seconds before the five-minute mark, un-mute yourself and start your video.
- At EXACTLY five minutes: ***Okay, that is time.***

Would one of you let (Child) know it's time to come back?

ADAPT SMART STUDY

7: MONITORING DISCUSSION

Time: 4 min.

- When the child returns, make sure s/he is sitting in the seat between the parents.
- Verify that the session is still recording.
- Check the screen to make sure the participants are properly framed. If they are not, make any needed adjustments.

(Child), please talk about a time in the LAST MONTH when you were with your friends without an adult. This doesn't necessarily mean that an adult wasn't around at all - only that they weren't hanging out with you and your friends. Do you have a time in mind that you could talk about?

1. IF THE CHILD CANNOT THINK OF A TIME

(***Parents***)***, do you have any ideas of what (Child) could talk about?***

2. IF THE PARENTS CANNOT THINK OF A TIME

You may offer the following suggestions (based on child age): playing at the playground at school, playing in the backyard, going to the mall, or playing in the neighborhood. Avoid the option of playing in the next room.

3. IF THE CHILD HAS NOT HAD ANY IN-PERSON PEER INTERACTIONS (WITHOUT AN ADULT)

(Child), is there a time when you were with your friends virtually without an adult, like online or on FaceTime or Zoom?

4. IF THE FAMILY STILL CANNOT THINK OF A TIME

The task must be skipped, and the reason for the missing data documented in the Child Interviewer Impressions survey.

STUDY

Once a topic has been confirmed: **(*Child*), *go into as much detail as you'd like, starting from the beginning and going to the end. Talk about where you were, who you were with, and what you were doing. (Parents), please first listen to (Child) and then comment or gather any other information you might be interested in.***

I'll come back in 4 minutes. Any questions?

Then you may begin.

- Start the timer.
- Click "Stop Video" and "Mute" on your Zoom screen.
- A few seconds before the four-minute mark, un-mute yourself and start your video.
- At EXACTLY four minutes: ***Okay, that is time.***

8: FUN FAMILY ACTIVITY

Time: 3 min.

- Verify that the session is still recording.
- Check the screen to make sure the participants are properly framed. If they are not, make any needed adjustments.

We are down to our last activity of the day! The last thing we'd like you to do is plan a fun family activity together. This can be any fun activity that you'd like to do but should be something you can do together within the next week or so. There are no right or wrong ways to do this because every family is different.

I'll come back in 3 minutes. Any questions?

Then you may begin.

- Start the timer.
- Click "Stop Video" and "Mute" on your Zoom screen.
- A few seconds before the three-minute mark, un-mute yourself and start your video.
- At EXACTLY three minutes: ***Okay, that is time.***

ADAPT SMART STUDY

I am going to stop the recording now, and we have just a couple of quick things to go over, and then you are all done for the day.

- Click the "Stop Recording" button. It will ask if you want to stop recording.
- Click "Yes."

POST-FITS WRAP-UP WITH THE FAMILY

1. Child Prize

(<u>Child</u>), if you remember at the beginning of today's appointment, I said you would get a small prize for participating today.

Your prize is the puzzle game that you played with your parents earlier today. You get to keep it, and there's a lot more cards inside the game board with puzzles for you to put together.

2. Interview Compensation

<u>Confirming ClinCard Possession</u>
Some participants who completed their T1 online surveys during our hiatus received their ClinCards during that time. Others were mailed their ClinCards in the packet containing their assessment paperwork. Review the compensation section of the Family Information form to see when the participants should have received their cards and confirm that each parent has possession of his or hers.

<u>Compensation Distribution</u>
(<u>Parents</u>), you will be compensated $50 for completing today's interview. Would you prefer that $25 to be loaded onto each of your cards or that the full $50 be loaded onto one of the cards?

Make note of the family's preference.

The funds will be loaded onto your study MasterCard(s) within 48 hours. The envelope that your MasterCard came in gives you all of the information you need to use the card, but there are a couple of things I want to highlight:

a. ***First, you'll want to hang onto this card throughout the study because we will load all of your future payments onto it.***
b. ***Second, if you don't use the card for six months, the company starts charging small inactivity fees each month, so you'll want to make sure you use the funds before then.***

ADAPT SMART STUDY

3. Childcare Compensation (if applicable)

Some families will need to hire childcare providers for their other child(ren) in order to participate in the assessment. If that is the case, the Site Coordinator will make note of it on the compensation section of the Family Information form.

As you discussed when scheduling your appointment, you will also be given $30 to help cover the cost of childcare for today's appointment. Would you prefer that $15 be loaded onto each of your cards or that the full $30 be loaded onto one of the cards?

Make note of the family's preference.

4. Online Survey Compensation

If the parent completed it prior to the interview:

You already have $25 loaded onto your card for the online survey you completed prior to today's appointment.

If the parent did NOT complete it prior to the interview:

We'd still like you to complete the online survey that we sent you when we scheduled your appointment. It would be great if could complete it as soon as possible, and we will load an additional $25 on your card once you do.

IF ONE PARENT COMPLETED THE SURVEY AND ONE PARENT DID NOT, MAKE SURE YOU ARE CLEAR ABOUT WHO IS BEING COMPENSATED AND WHO STILL NEEDS TO COMPLETE THE SURVEY.

RT STUDY

5. Next Steps

One of our staff members will be following up with you in the next week or so to let you know which version of the parent program your family will be attending and an approximate timeline for getting started.

6. Final Questions and Conclusion

Do you have any final questions for me? Answer any questions they may have and thank them for meeting with us today.

Click "End" in the lower right-hand corner, and then click "End Meeting for All."

POST-ASSESSMENT

- (If applicable,) call the Site Coordinator to report any self-harm or mandated reporting issues.

- Email the Site Coordinator to let him/her know:
  - The assessment has been completed
  - Any time-sensitive information, excluding that related to self-harm and mandated reporting (e.g. participant no longer has ClinCard and needs to be issued a new one)
  - If the family wants their assessment (and childcare, if applicable) payment put on a particular parent's card or split evenly across the two

  DO NOT PUT ANY IDENTIFYING INFORMATION IN THE EMAIL (NAMES, EMAIL ADDRESSES, ETC.)

> SAMPLE:
>
> Subject: 99999 T1 Assessment
>
> Dear NAME,
>
> 99999's T1 assessment is complete.
>
> - Dad asked that you resend the email with his link to the online survey.
> - There are otherwise no time-sensitive issues to report.
> - The family requested that both the $50 for the interview and $30 for childcare be put on Mom's ClinCard.
>
> Thanks!
>
> NAME

- Complete the Child Interviewer Impressions survey in Qualtrics.

ADAPT STUDY
